# AI guided discovery of a murine model of asymptomatic Alzheimer’s disease

**DOI:** 10.1186/s40478-026-02286-y

**Published:** 2026-04-04

**Authors:** Suborno Jati, Sahar Taheri, Satadeepa Kal, Subhash C. Sinha, Brian P. Head, Sushil K. Mahata, Debashis Sahoo

**Affiliations:** 1 Department of Pediatrics, University of California San Diego, 9500 Gilman Drive, MC 0703 Israni Biomedical Research Facility Room No 2119, La Jolla, CA 92093 USA; 2 Veterans Medical Research Foundation, 3350 La Jolla Village Drive (151A), San Diego, CA 92161 USA; 3 Feil Family Brain and Mind Research Institute, Helen and Robert Appel Alzheimer’s Disease Research Institute, Weill Cornell Medicine, 413 East 69th Street, New York, NY 10021 USA; 4 VA San Diego Healthcare System, 3350 La Jolla Village Drive, San Diego, CA 92161 USA; 5 Metabolic Physiology & Ultrastructural Biology Laboratory, Department of Medicine, University of California, San Diego (0732), 9575 Gilman Drive; Stein Clinical Research Building #207, La Jolla, CA 92093-0703 USA; 6 Department of Chemistry and Biochemistry, University of California San Diego, La Jolla, United States; 7 Department of Anesthsiology, University of California San Diego, La Jolla, United States; 8 Department of Computer Science and Engineering, University of California San Diego, La Jolla, United States

**Keywords:** Chromogranin A, Boolean implication, Systems biology, Asymptomatic AD, Aging, Sex-specific resilience, Preventive therapeutic strategies

## Abstract

Alzheimer’s disease (AD) is a progressive neurodegenerative disorder defined by extracellular deposition of amyloid-β (Aβ) plaques and intracellular accumulation of hyperphosphorylated Tau in neurofibrillary tangles (NFTs). Notably, approximately 20–30% of older individuals harbor substantial amyloid and Tau pathology yet remain cognitively intact, a clinically silent state referred to as asymptomatic Alzheimer’s disease (AsymAD). The biological basis of this cognitive resilience remains poorly understood, in large part due to the absence of mechanistic frameworks and preclinical models that dissociate neuropathology from cognitive decline. Here, we integrate systems-level Boolean network modeling with in vivo validation to define the transcriptomic logic of AsymAD and establish an experimentally tractable murine model of cognitive resilience. Boolean implication networks trained on large-scale human cortical RNA-sequencing datasets identified a robust, invariant AD gene signature that accurately stratified disease states across multiple independent cohorts. Reverse translation of this signature to transgenic mouse models revealed a striking dissociation between molecular pathology and behavioral outcome in Chromogranin A (CgA)–deficient PS19 mice (CgA-KO/PS19). Male CgA-KO/PS19 mice exhibited AD-like transcriptomic and neuropathological features in the prefrontal cortex while retaining intact learning and memory. Female CgA-KO/PS19 mice demonstrated even greater resilience, characterized by suppression of Tau aggregation and preservation of synaptic ultrastructure. Together, these findings establish a validated murine model of AsymAD and identify CgA as a modifiable molecular node linking neuroendocrine signaling, Tauopathy, and cognitive preservation. This integrative computational–experimental framework provides a scalable and generalizable platform for dissecting sex-specific mechanisms of cognitive resilience, identifying early biomarkers of disease trajectory, and enabling mechanism-guided development of preventive therapeutic strategies for AD.

**Supplementary Information:**

The online version contains supplementary material available at 10.1186/s40478-026-02286-y.

## Introduction

Alzheimer’s disease (AD) is a progressive and ultimately fatal neurodegenerative disorder characterized by extracellular deposition of amyloid beta (Aβ) plaques and intracellular accumulation of hyperphosphorylated Tau in neurofibrillary tangles (NFTs) [1, 2]. Although these pathological hallmarks have long been considered obligatory correlates of dementia, large neuropathological and epidemiological studies have revealed that approximately 20–30% of elderly individuals harbor substantial amyloid and Tau pathology yet remain cognitively intact until death [3–7]. This clinically silent state, termed asymptomatic Alzheimer’s disease (AsymAD), represents a biologically distinct and clinically meaningful form of cognitive resilience rather than a simple prodromal stage of overt AD [8–12].

Human studies of AsymAD have identified multiple protective features, including selective neuronal hypertrophy, preservation of synaptic architecture, and a distinct immunological milieu characterized by attenuated microglial activation and reduced pro-inflammatory signaling [13–16]. Notably, AsymAD cohorts are often enriched for females, underscoring sex as a critical biological modifier of disease trajectory and cognitive resilience [17–20]. Despite these convergent observations, the molecular programs that enable sustained cognitive function in the presence of substantial neuropathological burden remain poorly understood [21, 22]. Progress in this area has been limited by two fundamental challenges: (i) the lack of systems-level analytical frameworks capable of identifying invariant disease logic across heterogeneous human datasets, and (ii) the absence of preclinical models that faithfully recapitulate the dissociation between pathology and cognition that defines AsymAD.

To address the first challenge, we employed Boolean implication network modeling, a systems biology approach that identifies stable, directional gene–gene relationships conserved across individuals, disease stages, and datasets [21, 23]. Unlike traditional differential expression analyses, Boolean modeling captures invariant regulatory logic and is inherently robust to biological and technical heterogeneity. We hypothesized that this approach would enable identification of a core transcriptomic signature of AD that persists across datasets and disease stages, thereby providing a principled framework for reverse translation into experimentally tractable models.

Among the candidates emerging from this systems-level analysis, Chromogranin A (CgA)—a neuroendocrine secretory granule protein expressed in neurons and glia [24–28], has been implicated in AD vulnerability [29–31]. CgA levels are elevated in the cerebrospinal fluid (CSF) of AD patients [32, 33], correlate with Tau pathology, and localize to NFTs [29, 31]. In prior work, we demonstrated that genetic depletion of CgA in PS19 Tauopathy mice (CgA-KO/PS19) attenuated Tau pathology and improves survival [33]. These findings suggested that CgA may function as a molecular amplifier of Tau toxicity and raised the possibility that CgA deficiency could uncouple neuropathology from cognitive decline.

In the present study, we explicitly test this hypothesis by integrating Boolean Network Explorer (BoNE) [34] modeling of human transcriptomic data with in vivo immunohistochemical, molecular, and ultrastructural analyses in CgA-KO and PS19 transgenic mice. This computational-experimental strategy enabled us to (i) identify a robust and invariant AD gene signature, (ii) apply this signature across multiple Tauopathy mouse models, and (iii) uncover a sex-dependent murine model of AsymAD, in which male CgA-KO/PS19 mice exhibit preserved cognition despite pathological challenge. Notably, female mice show even greater resilience, with reduced Tau phosphorylation and preserved synaptic ultrastructure. By establishing a mechanistic link between systems-level disease logic and sex-specific resilience in vivo, this work provides a conceptual and experimental framework for understanding the molecular basis of AsymAD as well as advancing preventive therapeutic strategies for AD.

## Results

### Boolean network modeling identifies an invariant transcriptomic signature of Alzheimer’s disease

**Fig. 1:**
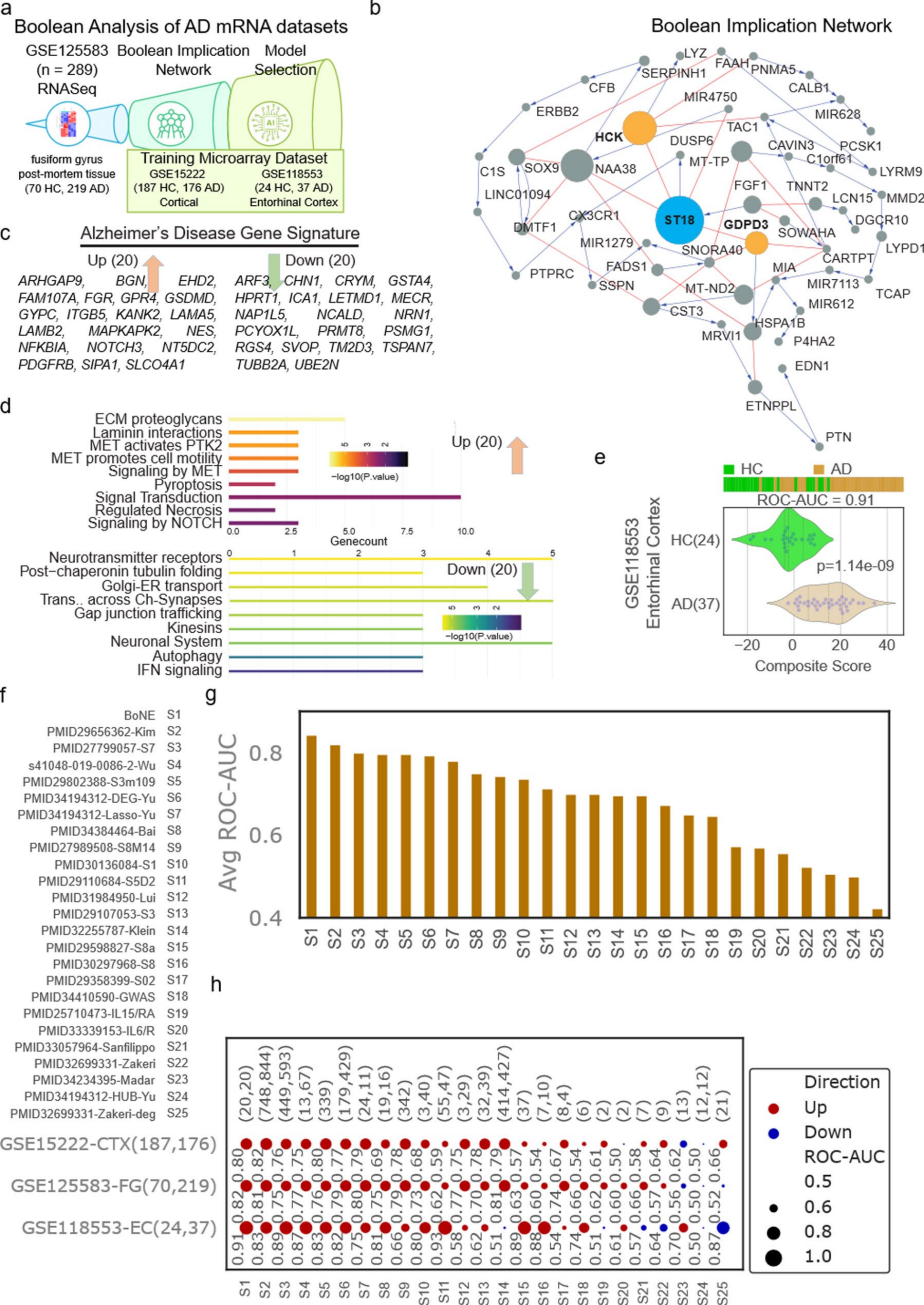
Boolean analysis of Alzheimer’s disease mRNA datasets. **a** Schematic representation of the Boolean analysis for Alzheimer’s disease (AD) datasets. **b** Network of clusters with representative gene name (size of circle = number of genes in the cluster) and Boolean implication relationships (red = hilo, blue – lolo). Top three clusters highlighted include differentially expressed genes between healthy control (HC) and AD brain samples. **c** AI/ML predicted gene signatures to distinguish HC and AD samples based on the network in panel b and three training datasets (GSE125583 Fusiform Gyrus, GSE15222 Cortical, GSE118553 Entorhinal Cortex). **d** Reactome analysis of the up (top)/down (bottom) regulated genes. **e** The composite score of the Boolean AD models was evaluated in GSE118553 Entorhinal Cortex, ordering of samples were visualized using bar plot and its distribution in each category is shown in violin plots. The ROC-AUC value and p-value from a two-sided unpaired T-test with unequal variance were computed to assess the Boolean model’s ability to distinguish between 24 HC (colored green) and 37 AD samples (colored orange) from the Entorhinal Cortex (GSE118553). **f** 24 publicly available gene signatures are ranked and compared to the trained Boolean AD model based on their average ROC-AUC values in distinguishing healthy control (HC) and Alzheimer’s disease (AD) samples across the three training datasets. **g** The results are presented in a bar plot, with the x-axis representing the ranked gene signatures and the average ROC-AUC values ordered from highest to lowest. **h** ROC-AUC analysis in bubble plots represent composite gene signature analyses of 24 previously published gene signatures and Boolean AD model (S1) in the three different training datasets. Up regulated gene signatures are colored red and down regulated gene signatures are colored blue. Bubble size corresponds to ROC-AUC, significance of p values is shown as *** (*p* < 0.001), ** (*p* < 0.01), * (*p* < 0.05), ‘.’ (*p* < 0.1)

To define a transcriptomic signature that captures invariant disease logic in Alzheimer’s disease (AD), we analyzed a large bulk RNA-sequencing dataset (GSE125583; 70 cognitively normal controls and 219 AD cases) using the Boolean Network Explorer (BoNE) framework. Unlike correlation-based approaches, BoNE constructs clustered Boolean implication networks based on directional gene–gene relationships, enabling identification of regulatory logic that is stable across individuals and datasets. The resulting AD network (“AD-net”; Fig. 1a–b) revealed coherent gene clusters corresponding to coordinated pathological processes.

To ensure robustness and generalizability, we refined the AD-net using three independent human datasets derived from distinct cortical regions (fusiform gyrus: GSE125583; frontal cortex: GSE15222; and entorhinal cortex: GSE118553). This analysis yielded a core 40-gene AD signature, consisting of 20 upregulated and 20 downregulated genes (Fig. 1c). The genes are listed in Supplementary Data, and their overlaps were compared in Supplementary Fig. 1. As can be seen from the histogram, no more than 10 genes overlap with other signatures and fraction overlapping are usually less than 0.1. Thus this 40-gene signature is distinct from the publicly available signatures. Reactome pathway enrichment analysis demonstrated that this Boolean-derived signature captured biological processes central to AD pathogenesis, including synaptic function, vesicle trafficking, and inflammatory signaling (Fig. 1d). Synaptic dysfunction is an early and robust feature of AD and correlates more strongly with cognitive decline than amyloid plaque burden, reflecting impairments in synaptic plasticity and neurotransmission [35, 36]. Alterations in vesicle trafficking pathways, including endosomal and lysosomal transport, are also well-established contributors to AD pathology and have been implicated in amyloid precursor protein processing and tau propagation [1, 2]. In addition, enrichment of inflammatory and immune-related pathways is consistent with extensive evidence linking microglial activation and chronic neuroinflammation to AD progression and neurodegeneration [13–16]. Together, these results indicate that the Boolean-derived signature captures core molecular mechanisms underlying AD rather than secondary or nonspecific transcriptional changes.

Composite scores derived from the upregulated and downregulated gene sets robustly discriminated AD cases from controls in the training datasets, achieving high receiver operating characteristic area-under-the-curve (ROC-AUC) values (Fig. 1e). Importantly, the Boolean AD signature consistently outperformed 24 previously published AD gene signatures across all training cohorts (Fig. 1f–h), demonstrating superior diagnostic accuracy and dataset invariance.

### Validation of boolean AD signature across independent human cohorts

**Fig. 2:**
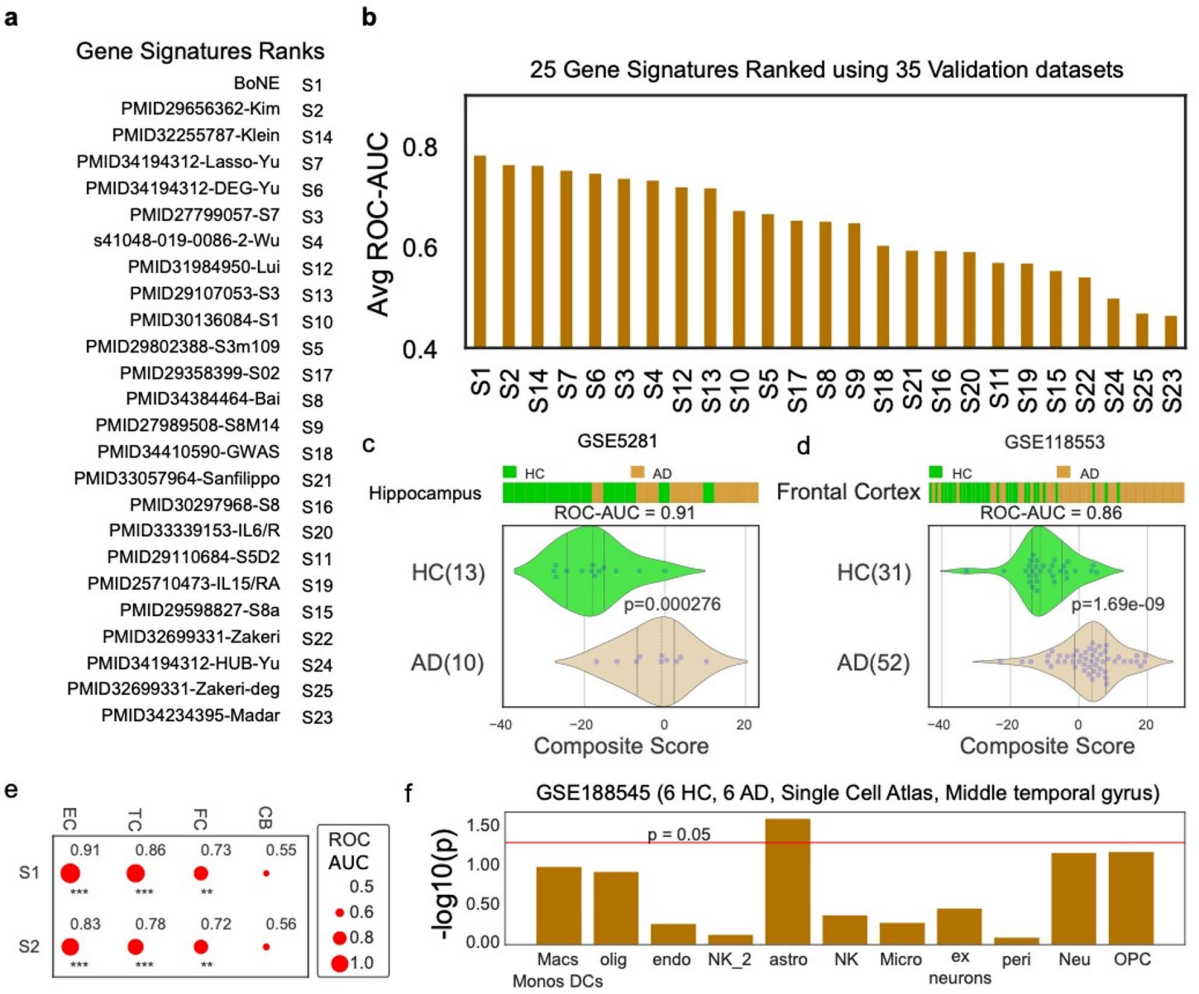
Validation of Boolean model in independent datasets. **a** The validation process involved ranking 24 publicly available gene signatures based on their average ROC-AUC values in distinguishing healthy control (HC) and Alzheimer’s disease (AD) samples across 35 independent validation datasets (See Supplementary Data). **b** The results are presented in a bar plot, with the x-axis representing the ranked gene signatures and the average ROC-AUC values ordered from highest to lowest. **c** The composite score of the Boolean AD models was evaluated, ordering of samples were visualized using bar plot and its distribution in each category is shown in violin plots. The ROC-AUC value and p-value from a two-sided unpaired T-test with unequal variance were computed to assess the Boolean model’s ability to distinguish between 13 HC (colored green) and 10 AD samples (colored orange) from the Hippocampus (GSE5281). **d** Bar, violin plot, ROC-AUC, and T-test were performed on another dataset, GSE118553, which included 31 HC and 52 AD samples from the frontal cortex. **e** A bubble plot was used to visualize the performance of two signatures, S1 and S2, in different brain regions, including the entorhinal cortex (EC), temporal cortex (TC), frontal cortex (FC), and cerebellum (CB). Bubble size corresponds to ROC-AUC, significance of p values is shown as *** (*p* < 0.001), ** (*p* < 0.01), * (*p* < 0.05), ‘.’ (*p* < 0.1). **f** Cell type specific differential expression patterns of the composite score are examined using a single cell RNASeq dataset on 6 HC and 6 AD samples from MTG tissue. Y-axis represents the -log10(p value). Red horizontal line represents the *p* = 0.05 threshold

To further assess robustness, we applied the Boolean AD signature to 35 independent human RNA-sequencing datasets spanning multiple brain regions and study designs. Across these datasets, the Boolean signature achieved the highest mean ROC-AUC values relative to all comparator signatures (Fig. 2a–d). Region-specific analyses recapitulated the known spatial progression of AD pathology, with elevated scores detected in the entorhinal cortex and hippocampus prior to widespread neocortical involvement (Fig. 2e). Together, these results confirm that the Boolean-derived signature captures conserved disease logic across cohorts, platforms, and anatomical regions.

It is important to characterize which cell types predominantly contribute to the Boolean AD signature. We examined the differential expression of AD model–identified genes across brain cell types using a single-cell brain atlas dataset (GSE188545; Fig. 2f) from the middle temporal gyrus (6 healthy control and 6 AD). Our analysis revealed that astrocytes exhibited significant transcriptional changes of the composite score of the Boolean AD model between healthy controls and AD samples, consistent with their involvement in disease pathology [37–39]. Multiple meta-analyses of late-onset AD (LOAD) genome-wide association studies (GWAS) have identified loci near genes highly expressed in astrocytes [40–42], suggesting that molecular pathways active in astrocytes contribute to AD risk and resilience.

To assess the robustness and generalizability of the Boolean AD model, we evaluated its performance in independent bulk RNA-seq (GSE285831) and pseudo-bulk snRNA-seq (GSE268609) datasets. The composite Boolean AD score significantly distinguished Alzheimer’s disease (AD) samples from healthy controls (HC) in both datasets (GSE285831: *p* = 0.0091, Supplementary Fig. 1b; GSE268609: *p* = 0.0181; Supplementary Fig. 1c). Consistent with model specificity, the Boolean AD score did not significantly differentiate HC from individuals with mild cognitive impairment, nor did it distinguish young from aged samples, indicating that the model preferentially captures molecular features associated with overt AD rather than normal aging or early-stage disease.

### Transition from human systems modeling to murine validation

Having established a robust and invariant AD transcriptomic signature in human datasets, we next sought to determine whether this signature could be reverse-translated to identify or validate preclinical mouse models that dissociate molecular pathology from cognitive impairment [38, 39]. This step was critical for bridging human systems-level findings with experimental neurobiology and for addressing the persistent lack of animal models that faithfully recapitulate AsymAD.

### Boolean modeling identifies a murine model of asymptomatic Alzheimer’s disease

**Fig. 3:**
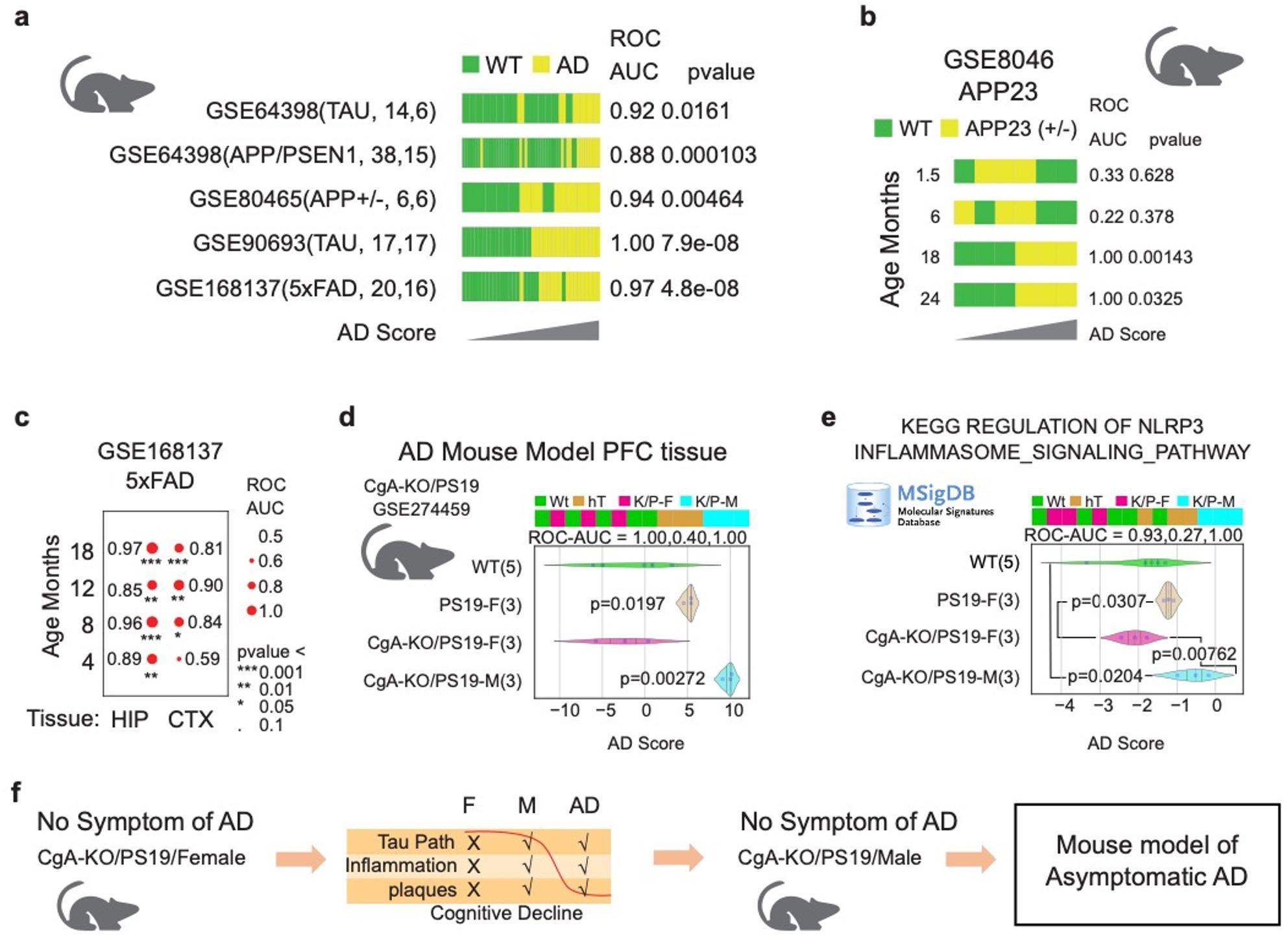
Discovery of mouse model of AsymAD. **a** Boolean AD model derived from human dataset was tested in five different mouse models of AD. Bar plots shows the sample ordering of the composite score from low to high. WT samples are color coded with green and AD samples are color coded with yellow. ROC-AUC and p value from a two-sided unpaired T-test with unequal variance is shown for each dataset. **b** WT and APP23 (+/-) AD samples (Hemi-forebrain) are compared in four different age groups (1.5, 6, 18, 24 months; GSE8046). The results are visualized using bar plots and ROC-AUC and p value from a two-sided unpaired T-test with unequal variance is shown for each dataset. **c** WT and 5xFAD AD samples are compared in four different age groups (4, 8, 12, 18 months; GSE168137) and two different tissues (Hippocampus and cortex). The results are visualized in a Bubble plot. Bubble size corresponds to ROC-AUC, significance of p values are shown as *** (*p* < 0.001), ** (*p* < 0.01), * (*p* < 0.05), ‘.’ (*p* < 0.1). **d** Bar, violin plot, ROC-AUC and T-tests were performed using the Boolean AD model composite score on a dataset with PFC samples from five different groups of mice (WT, PS19, CgA-KO/PS19 Female, CgA-KO/PS19 Male, CgA-KO). P values shown are based on two-sided unpaired T-tests with unequal variance comparing WT vs. the other four groups of mice. **e** Bar, violin plot, ROC-AUC and T-tests were performed using the composite score of genes described in the MSigDB GOBP INFLAMMASOME MEDIATED SIGNALING PATHWAY on a dataset with PFC samples from four different groups of mice (PS19, CgA-KO/PS19 Female, CgA-KO/PS19 Male, CgA-KO). P values shown are based on two-sided unpaired T-tests with unequal variance. **f** Boolean AD model discover that CgA-KO/PS19 Male mice PFC tissue have the AD disease states without any symptoms of the AD. Thus, this may represent the world’s first mouse model of the human AsymAD

We applied the human-derived Boolean AD signature to transcriptomic datasets from five widely used transgenic mouse models of AD (Fig. 3a). In established amyloid- and Tau-based models, Boolean scores accurately tracked disease progression and regional vulnerability. In the APP23 model (GSE8046), BoNE scores diverged between WT and AD mice only at 18 and 24 months (Fig. 3b). In the 5xFAD model (GSE168137), the hippocampus—but not cortex—showed robust disease stratification at early stages (Fig. 3c). Notably, when applied to prefrontal cortex transcriptomes from wild-type (WT), PS19 Tauopathy, and CgA-knockout PS19 (CgA-KO/PS19) mice, a striking dissociation between molecular pathology and behavioral outcome emerged.

Male CgA-KO/PS19 mice clustered transcriptomically with PS19 mice, exhibiting high Boolean AD scores indicative of an AD-like molecular state. Despite this, these mice retained intact spatial learning and memory in behavioral assays (Fig. 3d). In contrast, female CgA-KO/PS19 mice clustered with WT controls and exhibited additional protection at molecular and ultrastructural levels (Fig. 3d). This dissociation between transcriptomic pathology and preserved cognition mirrors the defining features of human AsymAD and identifies CgA-KO/PS19 male mice as a novel murine model of cognitive resilience.

### Preservation of synaptic vesicle architecture in female CgA-KO/PS19 mice

Sex differences in cognitive function and synaptic organization are well documented. While males often outperform females in spatial processing tasks, females generally exhibit advantages in verbal memory, verbal fluency, and speed of articulation [43, 44]. At the ultrastructural level, men have been reported to show higher synaptic density across cortical layers of the temporal neocortex [45]. Synapse loss and depletion of clear synaptic vesicles (CSVs; ~40–50 nm) and dense-core vesicles (DCVs; ~80–120 nm) are early hallmarks of AD and correlate strongly with cognitive decline [35, 46, 47].

**Fig. 4:**
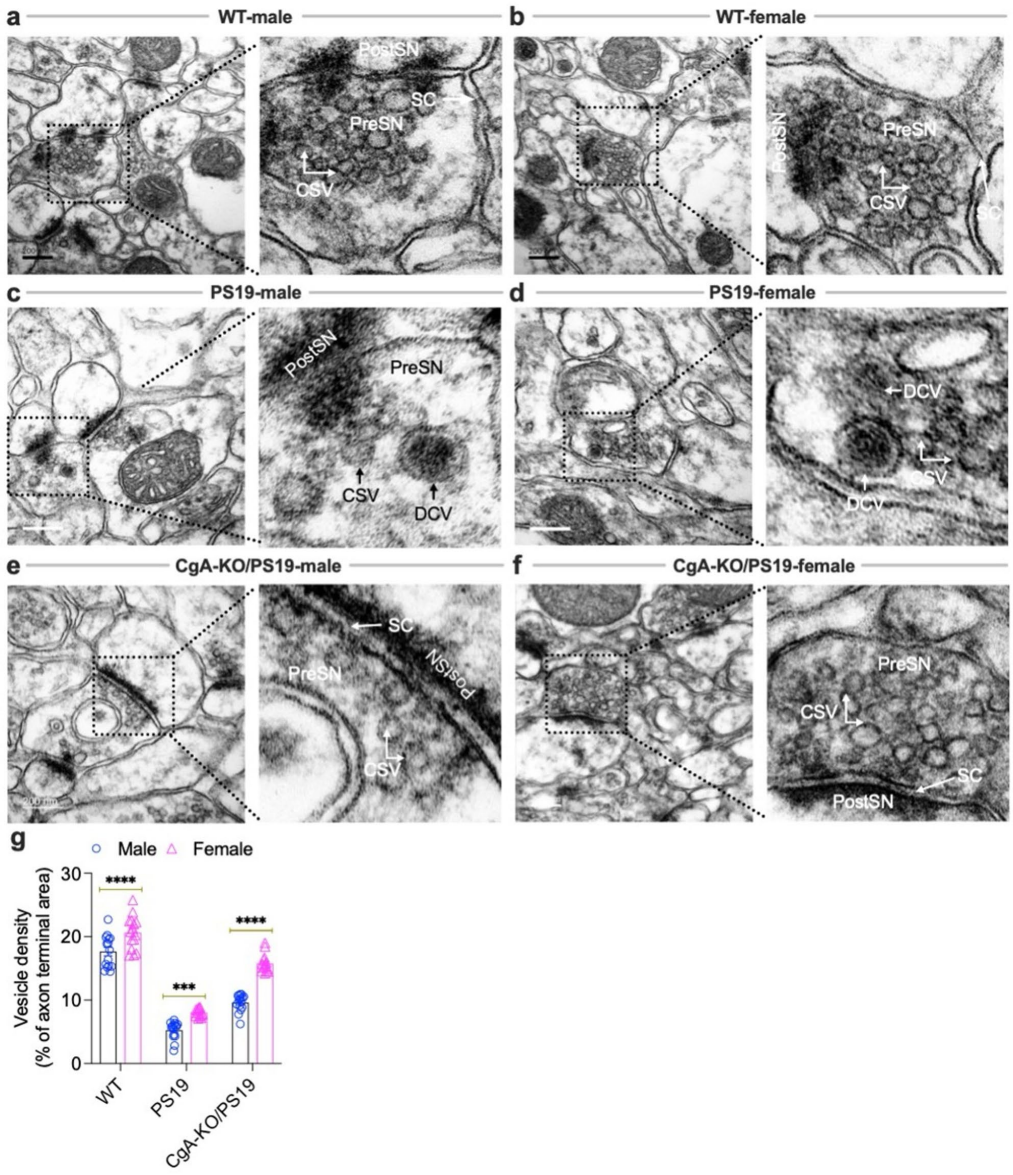
Ultrastructural analysis of synaptic vesicles in the pre-frontal cortices of WT, PS19, and CgA-KO/PS19 mice. Representative transmission electron micrographs of pre-frontal cortical synapses from wild-type (WT), PS19, and CgA-KO/PS19 mice of both sexes. **a** WT male and **b** female mice show abundant clear synaptic vesicles (CSVs) within presynaptic terminals (PreSN), indicative of intact synaptic architecture. **c** PS19 male and **d** female mice exhibit markedly reduced CSV density and disrupted synaptic organization, consistent with tauopathy-associated synaptic degeneration. **e** CgA-KO/PS19 male mice display a similarly diminished CSV pool as PS19 mice, suggesting persistent synaptic impairment. **f** In contrast, CgA-KO/PS19 female mice show restoration of CSV density comparable to WT, indicating preserved synaptic integrity. **g** Morphometric analysis of synaptic vesicles at the axon terminal to quantify vesicle density as a percentage of the total axon terminal area. Percent synaptic density was calculated by dividing the sum of the synaptic vesicle area with the area of the axon terminus and multiplied by 100. Note increased vesicle density in female WT, PS19 and CgA-KO/PS19 mice. Scale bars, 200 nm. PreSN: presynaptic terminal; PostSN: postsynaptic terminal; CSV: clear synaptic vesicle; DCV: dense-core vesicle; SC: synaptic cleft

Consistent with predictions from the downregulated BoNE gene signature—particularly those implicating impaired vesicle biogenesis and neurotransmission (Fig. 1d)—electron microscopy of hippocampal synapses revealed dense CSV clusters in WT males and females (Fig. 4a, b, g). Morphometric analysis revealed a significant sex-dependent effect on CSV density, with female mice exhibiting higher vesicle density than males in both WT and CgA-KO/PS19 genotypes (Fig. 4g), consistent with prior reports. Genotype analysis showed that PS19 mice exhibited a pronounced reduction in CSV density relative to WT controls in both sexes (Fig. 4c, d, g), consistent with the documented loss of presynaptic vesicle proteins, including synaptophysin and Rab3A, in AD brain tissue [48–50]. Notably, loss of CgA in CgA-KO/PS19 mice resulted in a robust increase in CSV density compared with PS19 mice in both males and females, with a particularly strong effect observed in females (Fig. 4g).

In line with our previous observations of elevated CgA and catecholamines in AD and PS19 brains, PS19 mice of both sexes exhibited a preponderance of DCVs (Fig. 4c, d). CgA-KO/PS19 male mice similarly showed a pronounced reduction in vesicle density (Fig. 4e, g). Remarkably, however, CgA-KO/PS19 female mice retained abundant CSVs (Fig. 4f, g). This preservation of presynaptic vesicle architecture likely contributes to their cognitive resilience and further validates the predictive power of the BoNE-derived transcriptomic signature.

### Sex-dependent accumulation of cortical neurofibrillary tangles

Multiple studies have demonstrated that women, particularly at symptomatic stages of AD, exhibit greater cortical NFT burden than men [51–53]. However, sex differences in tau pathology are increasingly recognized as stage- and context-dependent, with women also over-represented among individuals who remain cognitively intact despite substantial amyloid pathology (AsymAD).

**Fig. 5:**
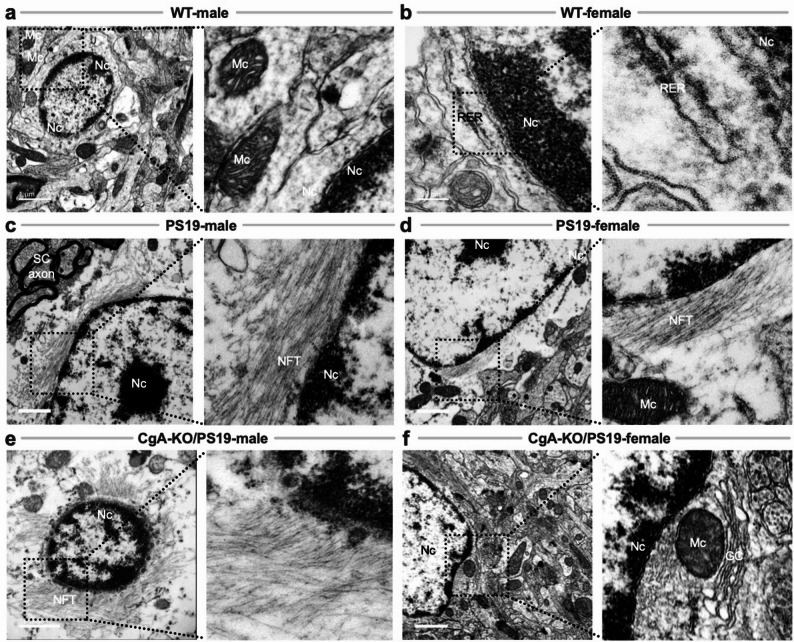
Neurofibrillary tangle (NFT) accumulation in dendritic regions of PS19 and CgA-KO/PS19 mice. Transmission electron microscopy (TEM) images of pre-frontal cortical neurons from WT, PS19, and CgA-KO/PS19 mice highlight the presence or absence of neurofibrillary tangles (NFTs) in dendritic regions. **a** WT male and **b** female pre-frontal cortices show healthy neuronal morphology with prominent nuclei (Nc), mitochondria (Mc), and intact rough endoplasmic reticulum (RER), with no evidence of NFT accumulation. **c** PS19 male and **d** female pre-frontal cortices exhibit extensive NFTs surrounding neuronal nuclei, consistent with advanced tauopathy. **e** CgA-KO/PS19 male mice retain dense NFT deposition in dendritic regions, similar to PS19. **F** In contrast, CgA-KO/PS19 female mice lack NFTs and display preserved neuronal ultrastructure comparable to WT controls. Scale bars: A, C, D, E, F = 1 μm; B = 200. nm. Nc: nucleus; Mc: mitochondria; RER: rough endoplasmic reticulum; NFT: neurofibrillary tangle; SC: synaptic cleft; GC: Golgi complex

Consistent with this complexity, NFTs were absent in WT mice (Fig. 5a–b) but abundant in PS19 males and females (Fig. 5c–d), indicating that female mice are not intrinsically protected from tau aggregation. CgA-KO/PS19 males closely mirrored PS19 pathology (Fig. 5e). In striking contrast, CgA-KO/PS19 females were largely devoid of cortical NFTs and exhibited preserved memory (Fig. 5f). These findings indicate that deletion of CgA selectively unmasks a female-specific protective mechanism that limits tau aggregation and neurotoxicity, modeling cognitive resilience analogous to human AsymAD rather than late-stage AD.

### Axonal and synaptic tau pathology mirrors dendritic trends

Tau pathology extends beyond dendrites to axonal and synaptic compartments and exhibits sex-dependent differences across disease stages [51–53]. Although women with symptomatic AD often exhibit greater cortical Tau burden [51–53], longitudinal imaging and postmortem studies indicate that female sex can confer resilience to Tau-mediated neurotoxicity under specific molecular contexts.

**Fig. 6:**
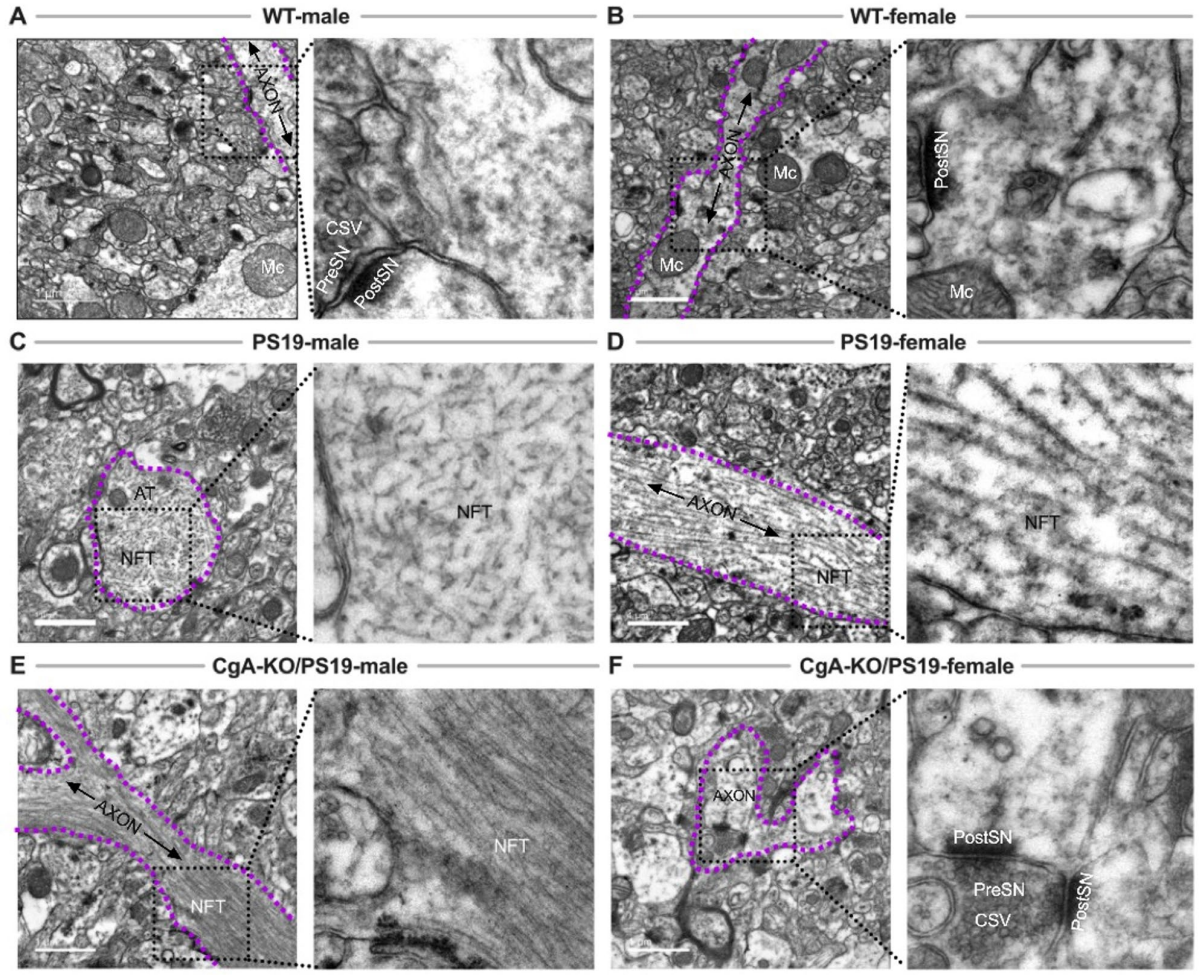
Neurofibrillary tangles (NFTs) in axons and axon terminals of PS19 and CgA-KO/PS19 mice. Transmission electron microscopy (TEM) images of pre-frontal cortical axons and axon terminals from WT, PS19, and CgA-KO/PS19 mice highlight NFT distribution in tauopathy. **a** WT male and **b** female pre-frontal cortices show normal axonal ultrastructure with no evidence of NFT accumulation. **c** PS19 male and **d** female mice exhibit dense filamentous NFTs within axons and axon terminals, characteristic of advanced tau pathology. **e** CgA-KO/PS19 male mice show NFT deposition comparable to PS19, indicating sustained tauopathy. **f** In contrast, CgA-KO/PS19 female mice lack detectable NFTs and exhibit preserved axonal morphology, mirroring WT. Scale bars: 1 μm. NFT: neurofibrillary tangle

Consistent with our dendritic analyses, electron microscopy revealed NFTs within axons of PS19 males and females and in CgA-KO/PS19 males (Fig. 6c-e). In contrast, axonal NFTs were absent in WT mice and in female CgA-KO/PS19 mice (Fig. 6a, b, f). Together with the absence of dendritic NFTs described above, these findings indicate a coordinated suppression of tau aggregation across neuronal compartments in female CgA-KO/PS19 mice, supporting a sexually dimorphic mechanism consistent with asymptomatic resilience rather than reduced disease prevalence.

### Suppression of misfolded tau species in female CgA-KO/PS19 mice

**Fig. 7:**
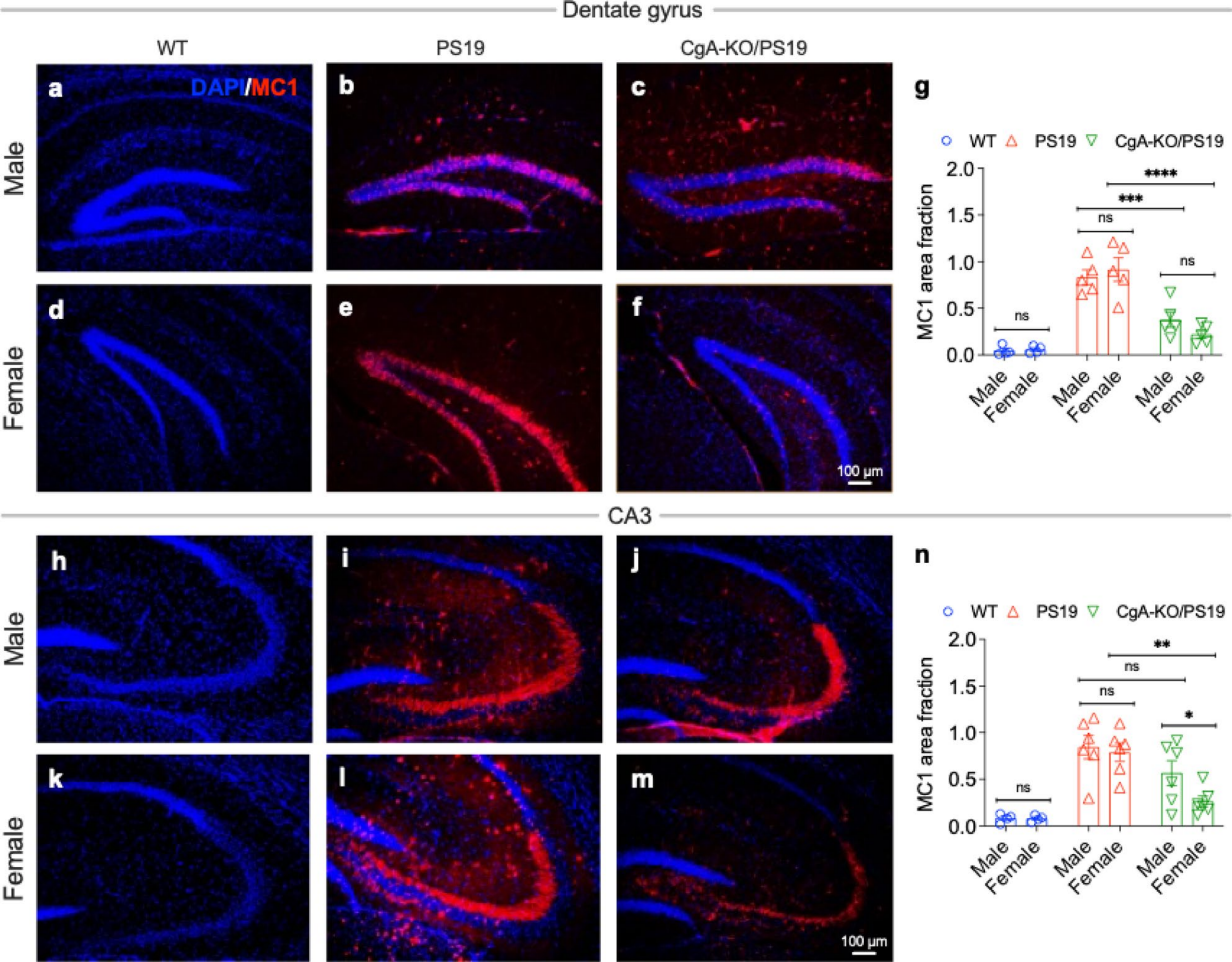
CgA deficiency reduces misfolded Tau aggregation in female PS19 mice. Representative hippocampal sections from the dentate gyrus (DG) region show MC1 immunostaining for misfolded Tau (red) with DAPI nuclear counterstain (blue) in **a** WT male, **b** PS19 male, **c** CgA-KO/PS19 male, **d** WT female, **e** PS19 female, and **f** CgA-KO/PS19 female mice. **g** Quantification of MC1-positive area fraction in the DG. Robust MC1 immunoreactivity is observed in PS19 mice of both sexes. While CgA-KO/PS19 males show a modest reduction compared with PS19 males, CgA-KO/PS19 females exhibit a marked decrease in MC1 signal. Representative sections from the CA3 region are shown in **h** WT male, **i** PS19 male, **j** CgA-KO/PS19 male, **k** WT female, **l** PS19 female, and **m** CgA-KO/PS19 female mice. **n** Quantification of MC1-positive area fraction in CA3 demonstrates a significant reduction in misfolded Tau burden in CgA-KO/PS19 females compared with PS19 females, whereas no significant difference is observed between PS19 and CgA-KO/PS19 males. Data are presented as mean ± SEM. Statistical analysis was performed using two-way ANOVA with post hoc multiple-comparisons testing. *p < 0.05, **p < 0.01, ***p < 0.001, ****p < 0.0001; ns, not significant. Scale bar: 100 μm

To determine whether this compartment-wide suppression of tau aggregation also extends to pathological tau conformations, we next quantified misfolded tau species using MC1 immunostaining. As expected, PS19 mice of both sexes displayed robust accumulation of misfolded tau relative to WT controls in the dentate gyrus (DG; up to ~ 18.5-fold increase; Fig. 7a, b, d, e, g) and CA3 (~ 10-fold increase; Fig. 7h, i, k, l, n). Strikingly, female CgA-KO/PS19 mice showed a marked reduction in MC1-positive tau in both hippocampal subregions, with decreases of ~ 23% in the DG (Fig. 7e, f, g) and ~ 33% in CA3 (Fig. 7l, m, n) compared with PS19 mice. In contrast, male CgA-KO/PS19 mice exhibited only a modest reduction in the DG (Fig. 7b, c, g) and no detectable reduction in CA3 (Fig. 7i, j, n). Together, these data demonstrate that CgA deletion suppresses tau misfolding in a sex-dependent manner. This finding is consistent with the Boolean network–derived transcriptomic predictions and aligns with the preserved cognitive performance observed specifically in female CgA-KO/PS19 mice.

## Discussion

In this study, we establish an experimentally validated murine model of AsymAD by integrating systems-level Boolean network modeling with immunohistochemical, molecular, and ultrastructural analyses. AsymAD—defined by the presence of hallmark AD pathology in the absence of overt cognitive impairment—affects an estimated 20–30% of cognitively intact elderly individuals [3–7]. Despite its clinical and biological significance, the mechanisms underlying cognitive resilience in AsymAD have remained poorly understood, due in large part to the absence of analytical frameworks capable of identifying invariant disease logic across heterogeneous human datasets and the lack of preclinical models that dissociate neuropathology from cognitive decline [6, 7, 21, 22, 54, 55].

By applying the BoNE to large-scale human transcriptomic datasets, we identified a core 40-gene AD signature that was robust across brain regions, cohorts, and experimental platforms [56–62]. This Boolean-derived signature consistently outperformed previously published AD gene sets in independent validation datasets, underscoring the advantage of Boolean implication modeling in capturing stable regulatory relationships rather than cohort-specific differential expression. Importantly, this approach enabled principled reverse translation from human molecular data to experimental mouse models [59], thereby establishing a scalable conceptual bridge between systems biology and in vivo neurodegeneration research.

When applied to transcriptomic datasets from transgenic mouse models, the Boolean AD signature accurately tracked disease progression in established amyloid- and Tau-based models. Most notably, its application to CgA-KO/PS19 mice revealed a striking dissociation between molecular pathology and cognitive outcome. Male CgA-KO/PS19 mice exhibited AD-like transcriptomic signatures and Tau-associated neuropathology while retaining intact spatial learning and memory, thereby recapitulating a defining feature of human AsymAD. Female CgA-KO/PS19 mice displayed an even greater degree of resilience, characterized by reduced Tau phosphorylation, absence of NFTs, preservation of synaptic vesicle ultrastructure, and attenuation of inflammatory signaling. Together, these findings establish male CgA-KO/PS19 mice as a biologically grounded model of cognitive resilience—rather than disease resistance per se—and provide experimental access to mechanisms that decouple neuropathology from cognitive decline.

These observations highlight sex as a critical biological variable in modulating vulnerability and resilience to neurodegeneration. Epidemiological and neuropathological studies consistently demonstrate that women bear a higher lifetime risk of AD and often exhibit greater Tau burden at symptomatic stages of disease [63, 64]. However, accumulating evidence indicates that sex differences in AD are stage- and context-dependent; during preclinical and early disease stages, women may demonstrate greater cognitive resilience—manifesting as better memory performance than men at comparable levels of AD pathology or biomarker burden—an advantage that diminishes as pathology advances [65–68]. Our findings strongly support this nuanced framework, suggesting that female-specific protective mechanisms operate early in disease progression but may be overcome during later symptomatic phases. Potential contributors include sex hormone signaling, sex chromosome dosage, and sex-specific immune, glial, and synaptic responses. Notably, our prior studies pooled male and female animals, likely obscuring these effects; the present work underscores the necessity of sex-stratified analyses in both computational and experimental AD research.

We further identify CgA as a modifiable determinant of disease vulnerability and resilience. CgA is elevated in the CSF of AD patients [69], correlates with Tau pathology, and localizes to NFTs [29, 31]. Prior studies demonstrated that genetic deletion of CgA attenuates Tauopathy and improves survival in PS19 mice [33]. Here, we extend those findings by demonstrating that CgA deficiency permits preservation of cognitive function despite the persistence of AD-like molecular signatures, indicating that CgA modulates downstream pathogenic processes that are not fully captured at the transcriptomic level alone. Given the established roles of CgA and its cleavage product Catestatin (CST: hCgA_352-372_) in catecholaminergic signaling [70–72], neuroimmune modulation [73–75], neurodegeneration [33, 76], and synaptic vesicle biology [77–79], CgA emerges as a central integrator of neuroendocrine and neuroimmune pathways relevant to AD progression.

Collectively, this work establishes a mechanistically grounded framework for studying cognitive resilience in AD and provides a scalable platform for interrogating sex-specific protective pathways, identifying early biomarkers of disease trajectory, and enabling mechanism-guided development of preventive therapeutic strategies. By decoupling neuropathology from cognition in vivo and anchoring this dissociation in invariant human disease logic, our findings shift the focus of AD research toward resilience mechanisms that may be therapeutically exploitable before irreversible neurodegeneration occurs.

### Limitations and future directions

Despite the strengths of this study, several limitations warrant consideration and, importantly, define clear directions for future investigation. First, our molecular and neuropathological analyses focused primarily on the hippocampus and prefrontal cortex, regions central to learning, memory, and executive function. However, other brain regions implicated early in AD pathogenesis—including the anterior cingulate cortex, basal forebrain, and locus coeruleus [37, 80]—were not examined. Incorporation of these anatomically and functionally distinct regions will be essential to determine the regional generalizability of the identified resilience mechanisms and to assess whether resilience is instantiated through shared or region-specific molecular programs.

Second, although we observed robust sex-dependent differences in cognitive resilience, the underlying biological mechanisms remain unresolved. Future studies will be required to systematically dissect the contributions of sex hormone signaling, sex chromosome dosage, epigenetic regulation, and sex-specific chromatin architecture to Tau pathology, synaptic preservation, and behavioral outcomes [19, 81]. Such analyses will be critical for distinguishing organizational versus activational effects of sex and for determining whether female-associated resilience mechanisms can be therapeutically engaged in both sexes.

Third, we acknowledge inherent limitations of data-driven and AI-based approaches, including dependence on input data quality, cohort composition, and feature representation [82–84]. While Boolean implication modeling is explicitly designed to capture invariant regulatory logic and mitigate biological and technical heterogeneity, complementary analytical strategies and prospective perturbation-based validation will be necessary to establish causality and refine mechanistic inference. Integration of experimental manipulation with systems-level predictions represents a key next step toward translating network logic into actionable therapeutic insight. Future work could address these limitations by incorporating larger and more diverse datasets, integrating multi-omics features, and exploring alternative AI approaches, including XGBoost, Random Forest, SVM-based methods, and advanced deep learning architectures such as convolutional neural networks (CNNs), to further enhance model performance and generalizability.

Finally, although BoNE identifies stable transcriptomic signatures, transcriptomic data alone cannot fully capture the multiscale processes underlying cognitive resilience. Integration with additional modalities—including proteomics, metabolomics, electrophysiological recordings, and cell-type–resolved analyses of neuronal and glial function—will be essential to bridge molecular logic with synaptic, circuit, and behavioral dynamics. Such multimodal integration will enable a more comprehensive understanding of how resilience is instantiated across biological scales and how it can be preserved or restored during disease progression.

## Conclusion

In summary, this study establishes a robust and experimentally validated framework for dissecting cognitive resilience in AD. By integrating Boolean logic–based systems modeling with a novel CgA-KO/PS19 mouse model, we define key molecular features of AsymAD and uncover sex-specific neuroprotective signatures that decouple neuropathology from cognitive decline. Within this framework, CgA emerges as both a biomarker and a modifiable determinant of disease susceptibility, positioning it as a promising node for mechanistic interrogation and therapeutic targeting.

More broadly, this scalable computational–experimental platform enables systematic and hypothesis-driven dissection of resilience mechanisms and reframes AD as a disorder characterized by divergent biological trajectories rather than inevitable cognitive failure. By shifting the focus from end-stage pathology to early, protective biology, this work lays a strong foundation for biomarker discovery, sex-aware intervention strategies, and the development of preventive approaches aimed at preserving cognitive function in at-risk populations. Collectively, these findings support a paradigm in which understanding and harnessing endogenous resilience mechanisms may represent a critical path forward for altering the course of AD.

## Methods

### Animals

All animal experiments were approved by the Institutional Animal Care and Use Committee (IACUC) at the University of California, San Diego (UCSD) and the VA San Diego Healthcare System, and were conducted in accordance with NIH guidelines and the ARRIVE reporting standards.

Chromogranin A knockout (CgA-KO) mice were originally generated on a mixed genetic background (50% 129/SvJ and 50% C57BL/6) using a *Cre*–*loxP* gene-targeting strategy to achieve congenital, whole-body deletion of the *Chga* gene [85]. These mice were subsequently backcrossed to C57BL/6J mice for seven generations to establish a congenic C57BL/6 background. To generate animals on a B6C3F1/J background, C57BL/6J CgA-KO mice were backcrossed to B6C3F1/J mice for four generations. CgA-KO mice were then crossed with PS19 heterozygous mice (B6C3F1/J background; Jackson Laboratory, stock #008169) to generate *CgA-KO/PS19* experimental animals [33].

All mice were housed in a temperature- and humidity-controlled facility under a 12-hour light/12-hour dark cycle with ad libitum access to food and water. Animals were maintained on a standard normal chow diet (NCD; 14% kcal from fat; LabDiet 5P00).

### Euthanasia and tissue collection

Mice were euthanized in accordance with IACUC-approved protocols and the 2020 AVMA Guidelines for the Euthanasia of Animals. Animals were placed in an induction chamber pre-filled with 3–5% isoflurane in oxygen (flow rate: 1–2 L/min) and monitored for loss of the righting reflex and absence of response to toe pinch, indicating attainment of a surgical plane of anesthesia. While under deep anesthesia, tissues were rapidly harvested, and euthanasia was completed by exsanguination.

### Genotyping

Mice were ear-tagged at weaning, and tail biopsies were collected for genotyping. Genomic DNA was extracted using the AccuStart Genotyping Kit (QuantaBio). PCR amplification was performed using AccuStart GelTrack PCR SuperMix according to the manufacturer’s instructions.

#### PS19 genotyping primers

- Forward (WT and mutant): 5′-TTG AAG TTG GGT TAT CAA TTT GG-3′.
- Reverse (WT): 5′-TTC TTG GAA CAC AAA CCA TTT C-3′.
- Reverse (Mutant): 5′-AAA TTC CTC AGC AAC TGT GGT-3′.

#### Chga genotyping primers

- Forward: 5′-GTA GCA TGG CCA CTA CCC AG-3′.
- Reverse: 5′-ATC CTT CAG AGC CCC TCC TT-3′.

### Building a comprehensive database of Alzheimer’s disease datasets

Publicly available microarray and RNA-sequencing datasets were obtained from the National Center for Biotechnology Information (NCBI) Gene Expression Omnibus (GEO) repository [86–88]. For Affymetrix microarray platforms, raw intensity files were normalized using Robust Multichip Average (RMA) [89]. For RNA-sequencing datasets lacking normalized values, expression levels were quantified as transcripts per million (TPM) [90] and log₂(TPM + 1) values were used for downstream analyses. In addition, we incorporated publicly available datasets normalized using alternative methods, including reads per kilobase per million mapped reads (RPKM) [91], fragments per kilobase per million mapped reads (FPKM) [92], TPM [93], and counts per million (CPM) [94]. For Affymetrix microarray data, RMA normalization was preferred over MAS 5.0 due to its superior performance in reducing technical variability and improving cross-sample comparability [95]. A complete list of training and validation datasets used in this study is provided in the Supplementary Data.

### StepMiner analysis

StepMiner is a computational algorithm designed to identify step-wise transitions in ordered or time-series data by fitting step functions that minimize sum-of-squared errors [96]. The algorithm evaluates all possible step positions in a series and identifies the location corresponding to the sharpest transition between low and high expression states. For each candidate step position, StepMiner computes the mean expression levels on either side of the step and applies an adaptive regression scheme to select the step that best fits the data.

For a dataset containing *n* observations (*X₁ … Xₙ*), fitted values (*Ŷi*) are estimated by assigning constant values before and after the step position *k*. The goodness of fit is assessed using an F-statistic defined as:

$$\:F=\frac{\sum\:_{i=1}^{n}({\widehat{X}}_{i}-\stackrel{\prime }{X}{)}^{2}/(m-1)}{\sum\:_{i=1}^{n}({X}_{i}-{\widehat{X}}_{i}{)}^{2}/(n-m)}$$

where *Xi* represents observed values, *Ŷi* represents fitted values, *m* denotes the degrees of freedom used in adaptive regression, and $\:\stackrel{\prime }{X}=\frac{1}{n}{\sum\:}_{j=1}^{n}{X}_{j}$is the mean expression level. The step position corresponding to the maximal F-statistic is selected as the optimal threshold, representing the gene expression switching point.

### Boolean analysis of gene expression data

Boolean analysis is a statistical framework that represents gene expression relationships using binary logic (high/low, 1/0) to infer invariant regulatory dependencies between gene pairs [34]. To perform Boolean analysis, continuous gene expression values were first discretized into binary states using the StepMiner algorithm. Expression values were sorted in ascending order, and a rising step function was fitted to determine a threshold. The midpoint of the step was used as the StepMiner threshold.

To reduce noise, a two-fold change margin (± 0.5 around the StepMiner threshold) was applied, and samples falling within this intermediate zone were excluded from Boolean inference. After discretization, each gene pair (A, B) was classified into four Boolean quadrants: (low, low), (low, high), (high, low), and (high, high), with corresponding counts denoted as *a₀₀*, *a₀₁*, *a₁₀*, and *a₁₁*.

Boolean implication relationships were inferred by identifying sparsely populated quadrants using BooleanNet statistics [34, 59]. The total number of samples was calculated as:

$$\:\mathrm{t}\mathrm{o}\mathrm{t}\mathrm{a}\mathrm{l}={a}_{00}+{a}_{01}+{a}_{10}+{a}_{11}$$

The number of samples in which gene A or gene B was in the low state was computed as:

$$\:{n}_{A,\mathrm{l}\mathrm{o}\mathrm{w}}={a}_{00}+{a}_{01},{n}_{B,\mathrm{l}\mathrm{o}\mathrm{w}}={a}_{00}+{a}_{10}$$

Expected counts ($\:\widehat{n}$) for each quadrant were calculated assuming independence between gene A and gene B:

$$\:\widehat{n}=\left(\frac{{n}_{A,\mathrm{l}\mathrm{o}\mathrm{w}}}{\mathrm{t}\mathrm{o}\mathrm{t}\mathrm{a}\mathrm{l}}\right)\left(\frac{{n}_{B,\mathrm{l}\mathrm{o}\mathrm{w}}}{\mathrm{t}\mathrm{o}\mathrm{t}\mathrm{a}\mathrm{l}}\right)\times\:\mathrm{t}\mathrm{o}\mathrm{t}\mathrm{a}\mathrm{l}$$

Quadrant sparsity was evaluated using a standardized statistic:

$$\:{S}_{ij}=\frac{\widehat{n}-n}{\sqrt{\widehat{n}}}$$

and an associated error probability:

$$\:{p}_{ij}=\frac{1}{2}\left(\frac{{a}_{ij}}{{a}_{ij}+{a}_{ik}}+\frac{{a}_{ij}}{{a}_{ij}+{a}_{lj}}\right)$$

A quadrant was considered sparse if S_ij_ > sThr and p_ij_ < pThr. Boolean implication relationships were defined based on the identity of the sparse quadrant(s). Symmetric relationships included Boolean equivalence (sparse off-diagonal quadrants) and Boolean opposition (sparse diagonal quadrants). Asymmetric relationships were inferred when a single quadrant was sparse, yielding implications such as A low ⇒ B high, A high ⇒ B low, and related forms.

Boolean implication relationships were identified using the criteria:

$$\:\mathrm{B}\mathrm{o}\mathrm{o}\mathrm{l}\mathrm{e}\mathrm{a}\mathrm{n}\:\mathrm{i}\mathrm{m}\mathrm{p}\mathrm{l}\mathrm{i}\mathrm{c}\mathrm{a}\mathrm{t}\mathrm{i}\mathrm{o}\mathrm{n}=({S}_{ij}>sThr,\hspace{0.25em}\:{p}_{ij}<pThr)$$

For the training dataset GSE125583, Boolean analysis was performed using thresholds *sThr = 3* and *pThr = 0.1*, consistent with previously validated BooleanNet parameters [95–97]. False discovery rates were estimated by random permutation of gene expression values within GSE125583, yielding FDR < 1 × 10^-5^under the selected thresholds.

### Boolean Network Explorer (BoNE)

Boolean Network Explorer (BoNE) is an integrated computational framework for constructing, visualizing, and interrogating networks of progressive molecular changes underlying disease or biological processes (Fig. 1a). BoNE operates in three sequential steps.

First, gene expression values from each dataset were discretized into binary states (high or low) using the StepMiner algorithm. Second, pairwise gene–gene relationships were classified into one of six possible Boolean implication relationships (BIRs), comprising two symmetric relationships (equivalent and opposite) and four asymmetric relationships (low ⇒ low, low ⇒ high, high ⇒ high, high ⇒ low), which were represented as Boolean implication statements. Unlike conventional network inference approaches (e.g., correlation-based or Bayesian models) that rely on symmetric linear dependencies, BIRs explicitly encode directionality and are robust to sample heterogeneity arising from differences in genotype, phenotype, disease severity, ethnicity, and experimental perturbations. Because all samples conform to the same underlying logical constraints, Boolean implication relationships are highly reproducible across independent datasets.

Third, genes with similar expression architectures—defined by sharing at least 50% of equivalence relationships with other genes—were grouped into clusters and organized into a higher-order network. In the resulting Boolean implication network, clusters of genes represent nodes and the dominant Boolean relationships between clusters define directed edges. BoNE enables unsupervised discovery of these structures while remaining agnostic to sample type or disease stage.

### Boolean implication network construction

A Boolean implication network (BIN) was constructed by identifying all statistically significant pairwise Boolean implication relationships among genes in the GSE125583 dataset (Fig. 1b). In the BIN, nodes correspond to genes and edges represent Boolean implication relationships. Equivalent and opposite relationships were represented as undirected edges, whereas the four asymmetric relationships (low ⇒ low, low ⇒ high, high ⇒ high, high ⇒ low) were represented as directed edges.

Prior to Boolean analysis, genes with insufficient dynamic range were excluded, as limited variability precludes reliable classification into high and low expression states. Dynamic range filtering was performed by examining the fraction of samples classified as high or low by StepMiner. Genes or probe sets with fewer than 5% of samples in either state were removed from further analysis, as previously described [34].

### Clustered Boolean implication network (CBIN)

To reduce network complexity and enhance interpretability, the BIN was transformed into a clustered Boolean implication network (CBIN) (Fig. 1b). Clustering was performed by grouping genes connected through Boolean equivalence relationships. While a naïve approach would construct connected components from equivalence edges, noise can introduce inconsistencies (e.g., inclusion of opposite relationships within the same component). To mitigate this, weak equivalence links were removed prior to clustering.

Specifically, a minimum spanning tree was computed for the equivalence graph, and a Jaccard similarity coefficient was calculated for each edge to quantify the overlap of shared connections between gene pairs. Edges with a Jaccard similarity coefficient < 0.5—indicating limited shared connectivity—were removed. Connected components formed after this filtering step were designated as clusters and constituted the nodes of the CBIN. Increasing the Jaccard similarity threshold yields more compact and internally correlated clusters.

Cluster size distributions were examined on a log–log scale to assess scale-free properties of the resulting network. The Jaccard similarity threshold was tuned to achieve approximately linear behavior on the log–log plot, consistent with scale-free organization.

Inter-cluster relationships were established by determining the dominant Boolean implication relationships between representative genes from each cluster. For each cluster, genes were ranked by the number of equivalence relationships within the cluster. Representative genes were selected from multiple ranks to sample cluster structure robustly. Boolean implication relationships between clusters were inferred by identifying the overwhelming majority relationship across sampled gene pairs. Additional implementation details are provided in the publicly released BoNE codebase.

CBIN edges were color-coded as follows: orange (low ⇒ high), dark blue (low ⇒ low), green (high ⇒ high), red (high ⇒ low), light blue (equivalent), and black (opposite).

### Composite score for clusters of genes

To generate a composite score for each cluster, gene expression values within each cluster were first normalized and averaged. Normalization was performed using a modified Z-score centered on the StepMiner threshold, according to the formula:

$$ \:{\mathrm{Normalized}}\:{\mathrm{expression}} = \frac{{\left( {{\text{expr - SThr - }}0.5} \right)}}{{3 \times \:{\mathrm{SD}}}} $$

where *expr* denotes the raw expression value, *SThr* the StepMiner threshold, and *SD* the standard deviation.

A weighted linear combination of cluster averages was then used to compute a composite score for each sample. Sample-wise scores were visualized using color-coded bar plots and violin–swarm plots (Fig. 1e). A noise margin corresponding to a two-fold change (± 0.5 around the StepMiner threshold) was applied to the composite score to account for variability inherent to expression discretization.

### Training AI models to predict Alzheimer’s disease states

Gene clusters were selected based on their ability to predict AD status in the training datasets. Within each cluster, genes were filtered according to predictive performance measured by receiver operating characteristic area under the curve (ROC–AUC) values, using thresholds of > 0.6 for upregulated genes and < 0.3 for downregulated genes across all three training datasets. The resulting sets of up- and down-regulated genes were ranked by *t*-statistics derived from the GSE125583 dataset, and the top 20 genes from each category were selected to construct the final predictive model. Upregulated and downregulated genes were assigned weights of + 1 and − 1, respectively, and combined to compute a composite score representing the trained AD model.

The training datasets integrated both bulk RNA-sequencing and microarray data derived from three distinct brain regions, enabling the model to capture biological and pathological heterogeneity across AD-relevant anatomical contexts. Model generalizability and robustness were further enhanced through incorporation of a Boolean Implication Network, which exploits invariant logical relationships that are conserved across heterogeneous datasets. Unlike correlation-based approaches, asymmetric Boolean implication relationships encode directionality and enable identification of temporally ordered biological processes. This framework has been successfully applied to characterize biological differentiation and disease progression processes, including B-cell differentiation using MiDReG [58, 59], bladder cancer differentiation [62], colon cancer and tissue differentiation [56, 57], prostate cancer differentiation [61], inflammatory bowel disease progression [60], and monocyte-to-macrophage differentiation [98].

Hyperparameters optimized during model training included the selection of gene clusters from the Boolean Implication Network, ROC–AUC cutoffs, and the number of genes retained based on ranked *t*-statistics. A 20-gene signature (10 upregulated and 10 downregulated genes) was insufficiently robust and generalizable, whereas inclusion of more than 40 genes (beyond 20 upregulated and 20 downregulated genes) did not yield further performance gains. Consequently, a 40-gene signature was selected as an optimal balance between robustness and model complexity. Model robustness and generalizability were further reinforced by restricting features to invariant Boolean implication relationships.

Model validation was performed using large, independent datasets spanning multiple brain regions, species, and age groups. Model performance was assessed using ROC–AUC metrics to discriminate AD from healthy control samples. A total of 35 validation datasets were assembled to benchmark the trained model against existing AD gene signatures, with GEO datasets stratified by brain region prior to analysis.

False discovery rates for BooleanNet statistics were estimated using randomized permutations of gene expression data. Because all possible gene pairs were evaluated, this analysis involved approximately 944 million gene–gene comparisons. Genes with low dynamic range were excluded prior to analysis to substantially reduce the number of tested pairs and improve statistical power. Model training was performed on an Ubuntu server equipped with four Intel^®^ Xeon^®^ CPU E3-1220 v2 processors (3.10 GHz) and 16 GB RAM, requiring approximately one hour of computation time. Model inference was computationally lightweight, enabling efficient application to large-scale transcriptomic datasets and potential clinical cohorts.

### RNA-seq library preparation and analysis

Total RNA was isolated from the prefrontal cortex of 8-month-old mice using the RNeasy Mini Kit (Qiagen), following the manufacturer’s protocol. RNA concentration and purity were assessed using a NanoDrop spectrophotometer, and RNA integrity was verified using an Agilent TapeStation 4200 system. RNA-sequencing libraries were prepared from 500 ng of total RNA using the KAPA mRNA HyperPrep Kit (Roche) with Unique Dual-Indexed Adapters (KAPA Biosystems). Libraries were PCR-amplified for 10 cycles, evaluated for quality using TapeStation, and quantified with a Qubit 2.0 fluorometer (Thermo Fisher Scientific).

Pooled libraries were sequenced on an Illumina NovaSeq 6000 platform using paired-end 100 bp reads at the UCSD Institute for Genomic Medicine (IGM) Core Facility.

### Immunohistochemistry

Mice were anesthetized with isoflurane and transcardially perfused with phosphate-buffered saline (PBS). Brains were fixed in zinc-formalin (Z-fix; Anatech) for 48 h, followed by cryoprotection in 30% sucrose for 72 h at 4 °C. Coronal brain Sect.  (30 μm thickness) were prepared using a sliding freezing microtome (Epredia) and stored at − 20 °C in cryoprotectant solution.

Sections (7–8 per mouse) spanning anterior to posterior hippocampal regions were selected for analysis. After extensive washing in PBS (6 × 10 min), sections were incubated with MC1 antibody (1:500; gift from Dr. Peter Davies) in PBS containing 0.4% Triton X-100 for 24 h at 4 °C. Sections were then washed (3 × 15 min in PBS) and incubated with fluorophore-conjugated secondary antibodies and DAPI (1:2000) for 1 h at room temperature. Following additional PBS washes, sections were mounted using Fluoromount-G (SouthernBiotech) and imaged with a Keyence BZ-X800 fluorescence microscope.

### Transmission electron microscopy (TEM)

Mice were deeply anesthetized and transcardially perfused with warm (37 °C) Hank’s Balanced Salt Solution (HBSS) containing calcium and magnesium, followed by fixation with 2.5% glutaraldehyde and 2% paraformaldehyde in 0.15 M sodium cacodylate buffer using a peristaltic pump, as previously described [99]. Brains were dissected, and hippocampal and cortical tissues were post-fixed in 1% osmium tetroxide (OsO₄) in 0.1 M cacodylate buffer.

Samples were en bloc stained with 2–3% uranyl acetate, dehydrated through a graded ethanol series, and embedded in resin. Ultrathin Sects. (50–60 nm) were collected and counterstained with 2% uranyl acetate and Sato’s lead solution. Images were acquired using a JEOL JEM-1400Plus transmission electron microscope equipped with a Gatan OneView 4k × 4k digital camera [75].

### Morphometric analysis

To minimize bias, electron micrographs were collected and analyzed in a randomized manner by three investigators blinded to experimental group assignment. A total of 25 electron micrographs were analyzed per group (*n* = 3 animals per group). Vesicle diameter and area were quantified using the line tool in ImageJ (NIH), while vesicle and axon terminal areas were traced using the freehand selection tool as previously described [99]. Vesicle density was calculated by dividing total vesicle area by the area of the corresponding axon terminal, as previously described [100].

### Statistical analyses

Gene signatures were used to classify samples into diagnostic categories, and classification performance was evaluated using receiver operating characteristic area under the curve (ROC–AUC) metrics. Gene signature–based classification was visualized using color-coded bar plots in combination with density plots or violin–swarm plots, as appropriate.

Statistical analyses were performed using R (version 3.2.3; 2015-12-10) unless otherwise specified. Differential expression analyses were conducted using the *scipy.stats.ttest_ind* function in Python (version 0.19.0), applying Welch’s two-sample *t*-test (unpaired, unequal variance, *equal_var* = FALSE) to accommodate unequal sample sizes. Multiple hypothesis testing was corrected using the Benjamini–Hochberg false discovery rate (FDR) procedure implemented in *statsmodels.stats.multitest.multipletests* (*fdr_bh*). Key results were independently validated using R statistical software (version 3.6.1; 2019-07-05).

Pathway enrichment analyses were performed using the Reactome database and associated algorithms⁷⁷. Reactome annotates signaling and metabolic components and organizes them into curated biological pathways and processes [101].

Survival analyses were conducted using Kaplan–Meier methods implemented in the *lifelines* Python package (version 0.14.6). Quantitative analyses of immunohistochemical and electron microscopy data were performed using GraphPad Prism software (version 10.5.0; San Diego, CA). For comparisons between two groups, unpaired two-tailed Student’s *t*-tests were applied. For comparisons involving more than two groups, one-way or two-way analysis of variance (ANOVA) was used, followed by Sidak’s multiple-comparison test where appropriate.

All data are presented as mean ± standard error of the mean (SEM). Statistical significance was defined as *P* < 0.05.

We selected the t-test rather than the Mann–Whitney U test because the assumptions underlying the t-test were reasonably satisfied in our dataset. Specifically, the t-test is designed to compare group means and is statistically more powerful when data are approximately normally distributed and measured on a continuous scale. In our analysis, we compute the composite score of a cluster of genes which is a weighted linear combination of gene expression values. We then compare the group means of this composite scores using the Welch’s Two Sample t-test (unpaired, unequal variance (equal_var = False), and unequal sample size). The distribution of composite score approximates normality because of the Central Limit Theorem. We tested this in one of our training datasets and demonstrated that the composite score is close to normal distribution (Supplementary Fig. 1d). Compared to non-parametric alternatives such as the Mann–Whitney U test, the t-test provides greater statistical power when its assumptions are met, allowing for more sensitive detection of differences between groups. Furthermore, the t-test directly evaluates differences in group means, which aligns with the biological and analytical objectives of our study. In contrast, the Mann–Whitney U test evaluates differences in rank distributions and does not specifically test differences in means unless distributional shapes are similar.

## Supplementary Information

Below is the link to the electronic supplementary material.

Supplementary Material 1.

Supplementary Material 2.

## Data Availability

All data are available in the main text or the supplementary materials. The codes are available at [https://github.com/sahoo00/ADnet](https:/github.com/sahoo00/ADnet). We created Boolean Lab Alzheimer’s Disease Benchmark (BoLAD benchmark) based on the training and validation datasets which can be downloaded from the link in the github page.
