# Supplementary material for: AI guided discovery of a murine model of asymptomatic Alzheimer’s disease
Source: Acta Neuropathol Commun. 2026 Apr 4;14:110. doi: 10.1186/s40478-026-02286-y (PMC13192062; doi:10.1186/s40478-026-02286-y)
Supplement: Supplementary file 2 — Supplementary Material 2. [file 40478_2026_2286_MOESM2_ESM.docx]

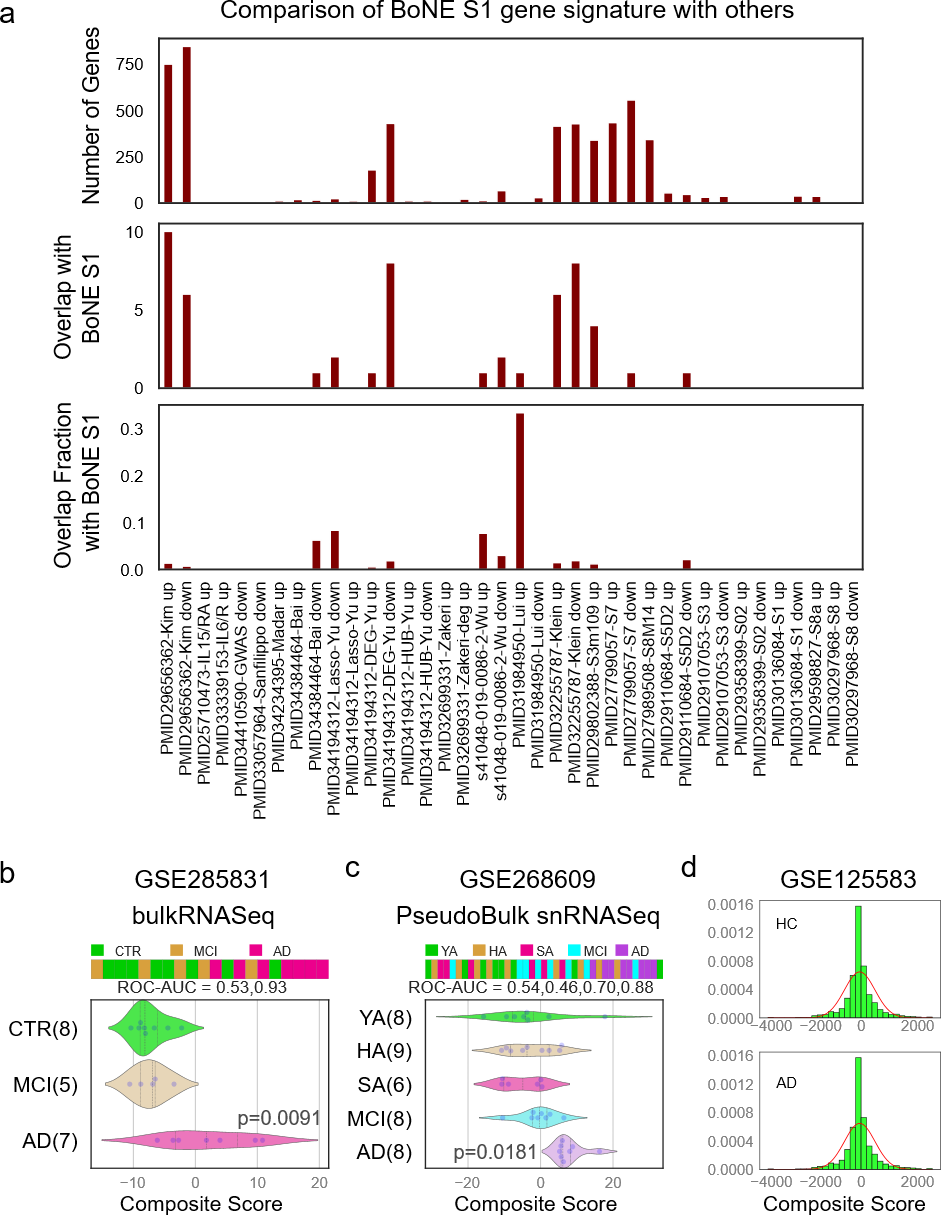


**Supplementary Figure 1. Comparison and validation of the Boolean AD model.**

(**a**) Comparison of the Boolean AD model (40-gene BoNE S1 signature) with previously published AD gene signatures to assess its uniqueness. Top: Number of up- and downregulated genes across 24 publicly available AD signatures. Middle: Number of overlapping genes between the BoNE S1 up-/downregulated genes and the corresponding up/down genes in each public signature. Bottom: Fraction of overlapping genes between the BoNE S1 up-/downregulated genes and the corresponding up/down genes in each public signature. (**b**) Validation of the Boolean AD model for predicting AD samples in a bulk RNA-seq dataset (GSE285831). CTR, healthy controls; MCI, mild cognitive impairment; AD, Alzheimer’s disease. The composite score of the Boolean AD model was evaluated in GSE285831 bulk RNASeq dataset, ordering of samples were visualized using bar plot and its distribution in each category is shown in violin plots. The ROC-AUC value and p-value from a two-sided unpaired T-test with unequal variance were computed to assess the Boolean model's ability to distinguish between 8 CTR (colored green), 5 MCI (colored orange) and 7 AD samples (colored pink) from postmortem human prefrontal cortex Brodmann area 9 (BA9) tissues. (**c**) Validation of the Boolean AD model for predicting AD samples in a recent snRNA-seq dataset (GSE268609). YA, young adult; HA, healthy adult; SA, super ager; MCI, mild cognitive impairment; AD, Alzheimer’s disease. The composite score of the Boolean AD model was evaluated in GSE268609 pseudobulk snRNA-seq dataset as described in panel b.

(**d**) Histogram and normal curve for randomly selected 20 up and 20 down regulated genes in the training dataset GSE125583. The composite signatures of these 40 random genes were used in the histogram for HC (top) and AD (bottom) separately.
